# The complete chloroplast genome of *Acer negundo* (Aceraceae)

**DOI:** 10.1080/23802359.2021.2008843

**Published:** 2021-12-10

**Authors:** Xudong Wang, Heyu Niu, Zhiyuan Huang

**Affiliations:** a School of Architecture, North China University of Water Resources and Electric Power, Zhengzhou, China; b College of Forestry, Henan Agricultural University, Zhengzhou, China

**Keywords:** *Acer negundo*, chloroplast genome, Illumina sequencing, genomic structure

## Abstract

*Acer negundo* L. is popular as ornamental shade trees. In this study, we sequenced, assembled and characterized the complete chloroplast genome of *A. negundo*. The genome sequence of *A. negundo* was 155,910 bp, consisting of a large single-copy region with 85,650 bp (LSC), a small single-copy region with 18,092 bp (SSC), and two inverted repeat regions with 26,084 and 26,090 bp (IRs). The GC content in the chloroplast genome of *A. negundo* was 37.9%. A total of 127 functional genes were predicted, including 83 protein-coding genes, 40 tRNA genes, and 4 rRNA genes. As shown in the phylogenetic tree, *A. negundo* was clustered into a monophyletic cluster.

Acer is a genus of Sapinduceae, including more than 124 species. Acer negundo is native to North America, for horticulture and landscaping purposes (Kowarik 2003; Nastasia 2016). It is an excellent street tree with colorful leaves and garden ornamentation. Its nectar of flowers is very rich, which is also a good nectar source plant. In order to clarify the taxonomical positions of *A. negundo* in Aceraceae, we applied the Illumina technology to sequence, assemble and annotated the whole chloroplast genome of *A. negundo*.

The fresh leaf samples of *A. negundo* was collected in Green Expo Garden, Zhengzhou, China (*A. negundo* N347517.1900; E1139290.5300). A specimen was deposited at the Herbarium of Henan Agricultural University (Heyu Niu, n32415@sina.com) under the voucher number AN-20-0716. The total genomic DNA was extracted from fresh leaves of *A. negundo* using a modified CTAB method (Doyle and Doyle 1987). Sequencing was performed with an Illumina HiSeq 2500 Platform (San Diego, CA, USA). The raw reads were generated by Illumina paired-end sequencing after removing adapters. The low quality sequences of raw reads used Fastp (https://github.com/OpenGene/Fastp) for quality control. Resultant clean reads were assembled using GetOrganelle pipeline v1.6.3a (https://github.com/Kinggerm/GetOrganelle) with the gene from *A. henryi* (GenBank accession no. NC_049163) as the reference sequence. The genome was automatically annotated by using the CpGAVAS2 pipeline (Shi et al. 2019) and start/stop codons and intron/exon boundaries were adjusted in Geneious 20.2.2 (https://www.geneious.com/).

The chloroplast genome sequence of *A. negundo* was submitted to NCBI, and the accession number was MT906791. The genome sequence of *A. negundo* was 155,910 bp in length, consisting of a large single-copy region with 85,650 bp (LSC), a small single-copy region with 18,092 bp (SSC), and two inverted repeat regions with 26,084 and 26,090 bp (IRs). The GC content in the chloroplast genome of *A. negundo* was 37.9%. The chloroplast genome of *A. negundo* contained 127 genes, including 83 protein-coding genes, 40 tRNA genes, and 4 rRNA genes.

The phylogenetic tree was constructed based on the genome sequences of *A. negundo* in RAxML v8.2 (Stamatakis 2006) with 1000 bootstrap replicates. A total of 16 species was used, including 14 Acer species and 2 Dipteronia species as outgroup (Zhou et al. 2017). Genome sequences were downloaded from the GenBank database and were aligned using MAFFT v7.0 (Katoh and Standley 2013).

As shown in the phylogenetic tree (Figure 1), the sixteen Acer species were organized into five clusters, which *A. negundo* was clustered into a monophyletic cluster, represents a distinct lineage of *A. negundo* that is probably spread throughout Asia. This result was similar to the previous phylogenetic trees based on chloroplast genome sequences of Acer (Yuan et al. 2020).

**Figure 1.**
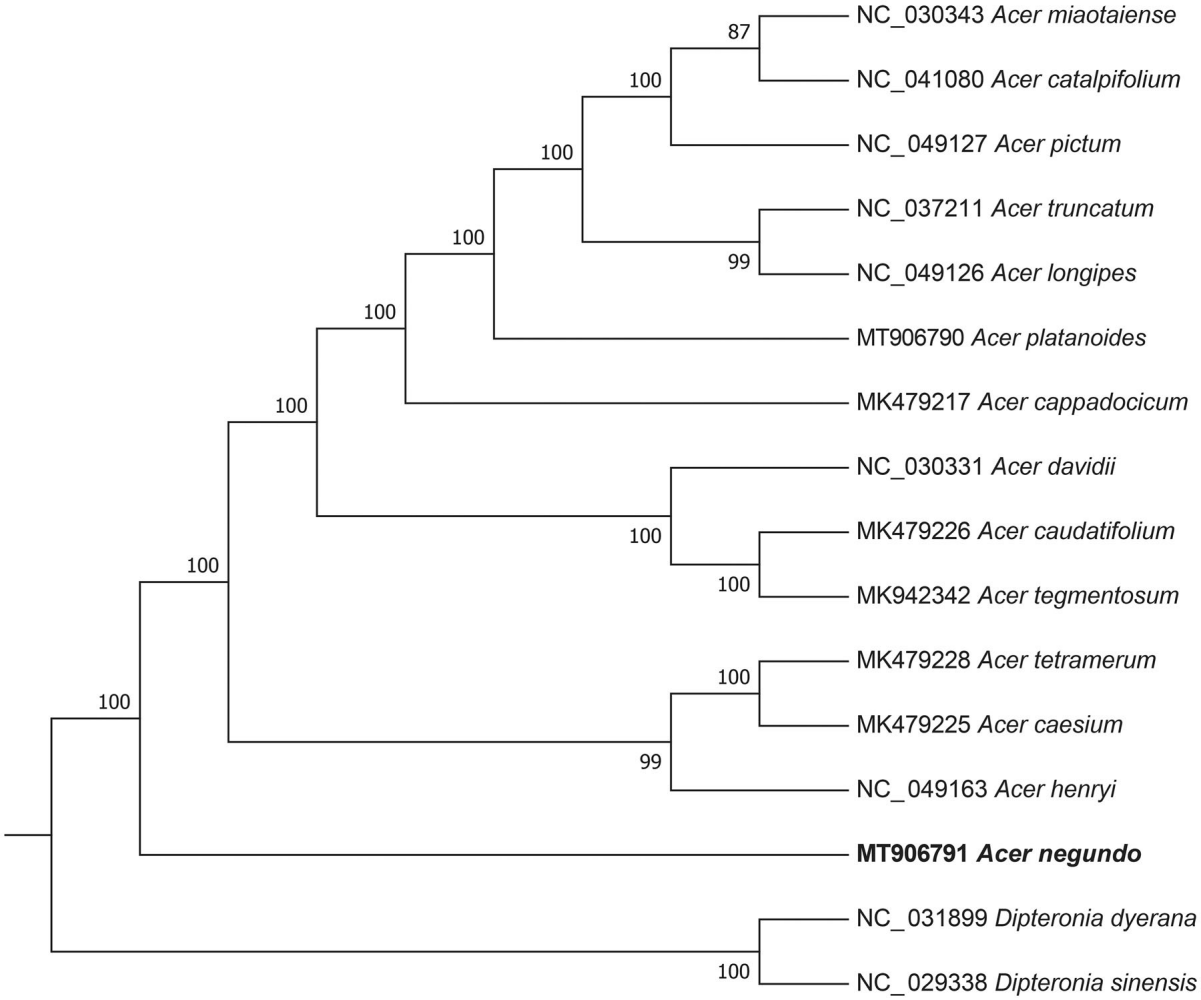
Maximum likelihood (ML) phylogenetic tree inferred from 14 plant chloroplast genomes. Numbers next to the branches are bootstrap support percentages.

## Data Availability

The data that support the findings of this study are openly available in the National Center for Biotechnology Information (NCBI) at https://www.ncbi.nlm.nih.gov/, reference number MT906791.The associated BioProject, SRA, and Bio-Sample numbers are PRJNA670186, SRA: SRS7542137, and SAMN16484703 respectively.
